# Supplementary material for: Deletion of wfs1 Impairs Oligodendrocyte Precursor Cells Dorsal Distribution and Myelination Through the wfs1-hmgcs1 Axis in Zebrafish
Source: Biology (Basel). 2026 Aug 16;15(16):1402. doi: 10.3390/biology15161402 (PMC13510151; doi:10.3390/biology15161402)
Supplement: Supplementary file 1 [file biology-15-01402-s001.zip › File S1-Transcriptome sequencing data and differentially expressed genes (DEGs) in wfs1-- zebrafish at 5 dpf/2.KEGG/wfs1KOvsWT/ALL/src/dre04530.html]

dre04530


- dre:792828

- Up regulated genes

792828\_1(7.34573383982123)

- Down regulated genes

792828(-4.67911285540239)

- dre:793158

- Up regulated genes

793158\_1(1.86160461223548)

- Down regulated genes

793158(-6.94943122774273)
- dre:556226

- Up regulated genes

556226\_1(6.36622858283894)

- dre:436718

- Up regulated genes

436718(1.34229427553561)

- Down regulated genes

436718\_1(-6.85571323128554)

- dre:321070

- Up regulated genes

101882911(3.50047007681359)

- dre:677750

- Up regulated genes

677750(1.37913343503877)

- dre:792699

- Down regulated genes

792699(-1.84389282152821)

- dre:100005566

- Up regulated genes

100005566(1.73719164936456)

- dre:559392

- Up regulated genes

559392\_1(5.40322756218621)

- Down regulated genes

559392(-2.91317811245432)

- dre:57934

- Up regulated genes

57934\_1(9.91304974129429)

- Down regulated genes

57934(-1.25539807424297)

- dre:100536545

- Down regulated genes

100536545(-1.61631046560488)

- dre:556298

- Down regulated genes

556298(-1.32024152429363)

- dre:57934

- Up regulated genes

57934\_1(9.91304974129429)

- Down regulated genes

57934(-1.25539807424297)

- dre:286739

- Up regulated genes

286739(1.28316470710399)

- Down regulated genes

286739\_1(-2.60068066374091)

- dre:569084

- Down regulated genes

569084\_1(-2.40277205964249)
- dre:81585

- Down regulated genes

81585(-1.74514319517014)
- dre:60635

- Down regulated genes

60635\_1(-1.07799587140666)
- dre:110438642

- Down regulated genes

110438642(-3.83724028917273)
- dre:793143

- Up regulated genes

793143(6.32099639946357)

- dre:677750

- Up regulated genes

677750(1.37913343503877)

- dre:793952

- Up regulated genes

793952\_1(1.28875020528538)

- dre:436718

- Up regulated genes

436718(1.34229427553561)

- Down regulated genes

436718\_1(-6.85571323128554)

- dre:793158

- Up regulated genes

793158\_1(1.86160461223548)

- Down regulated genes

793158(-6.94943122774273)
- dre:556226

- Up regulated genes

556226\_1(6.36622858283894)

- dre:431777

- Up regulated genes

431777(1.35055689210441)

- dre:445305

- Down regulated genes

445305\_1(-1.98867996431405)

- dre:57934

- Up regulated genes

57934\_1(9.91304974129429)

- Down regulated genes

57934(-1.25539807424297)

- dre:436718

- Up regulated genes

436718(1.34229427553561)

- Down regulated genes

436718\_1(-6.85571323128554)

- dre:797651

- Up regulated genes

797651\_1(1.84553757065454)

- Down regulated genes

797651(-1.27962292749959)
- dre:100005566

- Up regulated genes

100005566(1.73719164936456)

- dre:797651

- Up regulated genes

797651\_1(1.84553757065454)

- Down regulated genes

797651(-1.27962292749959)
- dre:100005566

- Up regulated genes

100005566(1.73719164936456)

- dre:569084

- Down regulated genes

569084\_1(-2.40277205964249)
- dre:81585

- Down regulated genes

81585(-1.74514319517014)
- dre:60635

- Down regulated genes

60635\_1(-1.07799587140666)
- dre:110438642

- Down regulated genes

110438642(-3.83724028917273)
- dre:793143

- Up regulated genes

793143(6.32099639946357)

- dre:569084

- Down regulated genes

569084\_1(-2.40277205964249)
- dre:81585

- Down regulated genes

81585(-1.74514319517014)
- dre:60635

- Down regulated genes

60635\_1(-1.07799587140666)
- dre:110438642

- Down regulated genes

110438642(-3.83724028917273)
- dre:793143

- Up regulated genes

793143(6.32099639946357)

- dre:569084

- Down regulated genes

569084\_1(-2.40277205964249)
- dre:81585

- Down regulated genes

81585(-1.74514319517014)
- dre:60635

- Down regulated genes

60635\_1(-1.07799587140666)
- dre:110438642

- Down regulated genes

110438642(-3.83724028917273)
- dre:793143

- Up regulated genes

793143(6.32099639946357)

- dre:569084

- Down regulated genes

569084\_1(-2.40277205964249)
- dre:81585

- Down regulated genes

81585(-1.74514319517014)
- dre:60635

- Down regulated genes

60635\_1(-1.07799587140666)
- dre:110438642

- Down regulated genes

110438642(-3.83724028917273)
- dre:793143

- Up regulated genes

793143(6.32099639946357)

- dre:792828

- Up regulated genes

792828\_1(7.34573383982123)

- Down regulated genes

792828(-4.67911285540239)

- dre:445305

- Down regulated genes

445305\_1(-1.98867996431405)

- dre:57934

- Up regulated genes

57934\_1(9.91304974129429)

- Down regulated genes

57934(-1.25539807424297)

- dre:100005566

- Up regulated genes

100005566(1.73719164936456)

- dre:100005566

- Up regulated genes

100005566(1.73719164936456)

- dre:100005566

- Up regulated genes

100005566(1.73719164936456)

- dre:792699

- Down regulated genes

792699(-1.84389282152821)

- dre:337226

- Up regulated genes

337226\_1(4.74603512488049)

- Down regulated genes

337226(-2.98693262894109)
- dre:798632

- Up regulated genes

798632(1.33935841885554)

- dre:321070

- Up regulated genes

101882911(3.50047007681359)

- dre:677750

- Up regulated genes

677750(1.37913343503877)

- dre:793158

- Up regulated genes

793158\_1(1.86160461223548)

- Down regulated genes

793158(-6.94943122774273)
- dre:556226

- Up regulated genes

556226\_1(6.36622858283894)

- dre:559392

- Up regulated genes

559392\_1(5.40322756218621)

- Down regulated genes

559392(-2.91317811245432)

Close
